# Vacuum-assisted evaporative concentration combined with LC-HRMS/MS for ultra-trace-level screening of organic micropollutants in environmental water samples

**DOI:** 10.1007/s00216-019-01696-3

**Published:** 2019-03-11

**Authors:** Jonas Mechelke, Philipp Longrée, Heinz Singer, Juliane Hollender

**Affiliations:** 1 Eawag, Swiss Federal Institute of Aquatic Science and Technology, 8600 Dübendorf, Switzerland; 2 Institute of Biogeochemistry and Pollutant Dynamics, ETH Zürich, 8092 Zürich, Switzerland

**Keywords:** Multiresidue analysis, PMOC, Large-volume injection, LC-HRMS, Non-target screening, Orbitrap

## Abstract

Vacuum-assisted evaporative concentration (VEC) was successfully applied and validated for the enrichment of 590 organic substances from river water and wastewater. Different volumes of water samples (6 mL wastewater influent, 15 mL wastewater effluent, and 60 mL river water) were evaporated to 0.3 mL and finally adjusted to 0.4 mL. 0.1 mL of the concentrate were injected into a polar reversed-phase C18 liquid chromatography column coupled with electrospray ionization to high-resolution tandem mass spectrometry. Analyte recoveries were determined for VEC and compared against a mixed-bed multilayer solid-phase extraction (SPE). Both approaches performed equally well (≥ 70% recovery) for a vast number of analytes (*n* = 327), whereas certain substances were especially amenable to enrichment by either SPE (e.g., 4-chlorobenzophenone, log*D*_ow,pH7_ 4) or VEC (e.g., TRIS, log*D*_ow,pH7_ − 4.6). Overall, VEC was more suitable for the enrichment of polar analytes, albeit considerable signal suppression (up to 74% in river water) was observed for the VEC-enriched sample matrix. Nevertheless, VEC allowed for accurate and precise quantification down to the sub-nanogram per liter level and required no more than 60 mL of the sample, as demonstrated by its application to several environmental water matrices. By contrast, SPE is typically constrained by high sample volumes ranging from 100 mL (wastewater influent) to 1000 mL (river water). The developed VEC workflow not only requires low labor cost and minimum supervision but is also a rapid, convenient, and environmentally safe alternative to SPE and highly suitable for target and non-target analysis.

**Electronic supplementary material:**

The online version of this article (10.1007/s00216-019-01696-3) contains supplementary material, which is available to authorized users.

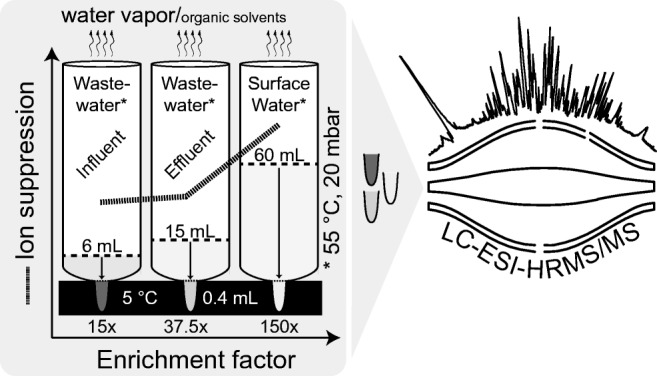

## Introduction

Organic contaminants (OCs) are constantly emitted into the aquatic environment with urban wastewater (WW), industry, and agriculture as the major sources [1]. Their potential (eco-)toxicological risk to humans, aquatic organisms, or whole ecosystems [2, 3] at the nanogram to microgram per liter level has caused extensive research activities over the last decades. Relevant nonpolar OCs are largely known, widely monitored, and regulated [4] but this barely applies to polar OCs that are highly mobile in the aquatic environment. Polar OCs, if persistent and widely emitted, have a significant potential to accumulate in the water cycle [5]. They are collectively referred to as PMOCs, i.e., persistent mobile OCs, with the anti-diabetic drug metformin being one prominent example along with its transformation product (TP), guanylurea.

Monitoring and regulation gaps are both linked to underlying analytical issues, i.e., polar OCs have the potential to go unnoticed as they are hardly amenable to state-of-the art liquid chromatography mass spectrometry (LC-MS) workflows currently and widely used for multiresidue trace organic analysis. These workflows often rely on pre-concentration by offline solid-phase extraction (SPE) with a single conventional sorbent material (e.g., C8, C18, mixed-mode) and LC on a reversed-phase (RP) stationary phase column [6–8]. While this combination is applicable to a wide range of moderately polar to nonpolar OCs, its suitability for highly polar OCs is limited [5]. Recent approaches that bypass or minimize this shortcoming include the following: (i) vacuum-assisted evaporative concentration (VEC) to dryness with subsequent hydrophilic interaction liquid chromatography (HILIC) [9], (ii) freeze-drying with subsequent mixed-mode LC [10], (iii) freeze-drying followed by (HILIC)-SPE and serial RPLC-HILIC or supercritical fluid chromatography (SFC) on a HILIC column [11], (iv) mixed-bed multilayer SPE optimized for retention of polar OCs with subsequent polar RPLC [9, 12–14], or (v) large-volume direct injection [15, 16]. Apart from these methods, chromatographic retention of polar OCs can additionally be enhanced by ion chromatography (e.g. [17, 18]), two-dimensional LC approaches (e.g. [19–21]), or parallel LC, e.g., HILIC parallel to RPLC with post column combination of eluents [22].

Established workflows for multiresidue trace analysis of very polar OCs are few but even fewer generic workflows exist for the simultaneous analysis of very polar to nonpolar OCs, especially when a fast, automated, reproducible, and (ultra-)sensitive analysis from a small sample volume is required. The aim of this work was to develop such workflow that covers the enrichment of OCs from different aqueous environmental matrices. The approach employs a single enrichment step using VEC, followed by large-volume injection (LVI) and chromatography on a polar RPLC column, coupled to high-resolution tandem mass spectrometry (HRMS/MS) via an electrospray ionization (ESI) interface. The major advantage of VEC is to bypass potential SPE pitfalls such as limited sorption capacity, analyte break-through during sample loading, unwanted elution of analytes during the wash step, incomplete analyte elution during the elution step, loss of analytes during the drying step, and analyte loss during nitrogen blow-down of the SPE extract. In contrast with other VEC approaches (e.g., see (i) above [9, 23, 24]), evaporation was not performed to dryness but to a residual volume of roughly 0.3 mL to avoid irreversible precipitation. To identify VEC workflow limitations especially for highly polar and nonpolar OCs, the workflow was validated for the enrichment of 590 substances with log*D*_ow_,_pH7_, i.e., the pH-dependent octanol-water distribution coefficient at pH 7, between − 14 (highly polar) and 8 (nonpolar). The aqueous environmental matrices included in this study ranged from a seemingly simple matrix (river water) to highly complex and “dirty” matrices such as wastewater influent (IWW) and effluent (EWW). To our knowledge, this is the first time that VEC was tested and validated for a large and diverse suite of OCs.

## Materials and methods

### Chemicals

Reference standards (STD) and isotope-labeled internal standards (IS) were purchased from CDN Isotopes (Canada), Dr. Ehrenstorfer (Germany), HPC Standards (Germany), LGC Standards (Switzerland), Molcan (Canada), MolPort (Latvia), Monsanto (Belgium), Novartis (Switzerland), Riedel-de-Häen (Germany), Sigma-Aldrich (Switzerland), or Toronto Research Chemicals (Canada) at purities ≥ 95% (analytical grade). NANOpure™ water (NPW) was generated using a lab water purification system (D11911, Barnstead/Thermo Scientific, USA). Methanol (MeOH) and ethanol were of LC-MS grade (Optima™, Fisher Scientific, Switzerland), ammonia (25% by weight) and formic acid of analytical grade (≥ 98%, Merck, Germany), and ethyl acetate of HPLC grade (99.8%, Sigma-Aldrich, Switzerland). STD and IS stock solutions (1 or 0.1 mg/mL) were prepared in appropriate solvents and combined as mixtures. These mixtures were then combined as spike solutions and subsequent dilutions were made in ethanol. An exhaustive substance list can be found in the Electronic Supplementary Material (see ESM 2 Table S6).

### Sample collection

Wastewater samples were taken at Wüeri wastewater treatment plant in Regensdorf, Switzerland. Sampling points were post primary clarification (IWW) and after the biological treatment (EWW). Surface water (SW) was collected at Chriesbach, Switzerland. All waters were grab sampled on February 24, 2016, and stored at 4 °C until use the following day.

### Vacuum-assisted evaporative concentration

SW and WW samples were equilibrated to room temperature, shaken thoroughly, and left to stand for 30 min to allow for settling of particles. Hence, no sample filtration or other sample treatment was applied prior to evaporation to minimize sample manipulation (see the “VEC workflow implications” section). Assuming water density at 20 °C, IWW (6 mL), EWW (15 mL), SW (60 mL), and NPW (60 mL, for the preparation of calibration standards) were carefully decanted and weighed into BUCHI™ glass vials (0.3 mL residual volume, 046069, BÜCHI Labortechnik AG, Switzerland). Depending on the validation experiment, samples were fortified with STD prior to VEC, after VEC, or not at all. Likewise, IS were spiked prior or after VEC. Water samples and calibration standards were then evaporated at 55 °C, 20 mbar and 200 to 300 orbital movements per minute using a vacuum-assisted evaporation system (see ESM 1 Fig. S1, Syncore® Analyst R-12, BÜCHI Labortechnik AG, Switzerland). Depending on sample size, parallel evaporation from up to 12 glass vials down to a residual volume of approx. 0.3 mL lasted 240 min (60 mL) or 80 min (≤ 15 mL) (see ESM 1 Table S1). A manual glass vial wall rinse involving 2 × 0.75 mL MeOH followed by 1 mL NPW was implemented after 210 min or 50 min, respectively, even though the device was operated with flushback module. Sample concentrates were transferred to flat bottom glass inserts (0.5 mL, 110506, BGB Analytik AG, Switzerland), visually adjusted to 0.4 mL using NPW, cooled down to 4 °C, and centrifuged at 10,621*g* for 4 min at room temperature (5427 R, Eppendorf, Switzerland). During centrifugation, glass inserts were kept inside microcentrifuge tubes (0030120094, Eppendorf, Switzerland). Glass pipettes were used to transfer supernatants to conical glass inserts (0.35 mL, 110502, BGB Analytik AG, Switzerland). The latter were kept in 2 mL amber glass LC vials at 4 °C until analysis. Enrichment factors were 15 (IWW), 37.5 (EWW), and 150 (NPW/SW).

### Solid-phase extraction

Prior to SPE, SW and WW samples were adjusted to pH 6.5 by adding ammonium acetate buffer (1 M, 1 mL), formic acid, and ammonia and subsequently filtered through glass fiber filters (GF/F, Whatman, UK). Depending on the validation experiment, filtered IWW (100 mL), filtered EWW (250 mL), filtered SW (1000 mL), and (unfiltered) NPW (1000 mL) were fortified with STD either prior to SPE, after SPE, or not at all, IS were added after SPE (“Absolute recovery of VEC and SPE step” section). SPE was performed over a cartridge containing 200 mg Oasis HLB (Waters, USA) as a top layer, a 350-mg mid layer of a 1:1:1.5 (*w*/*w*/*w*) mixture Strata X-AW, Strata X-CW (both: Phenomenex, USA), and Isolute ENV+ (Biotage AB, Sweden), and a 200-mg bottom layer ENVI-carb™ (Supelco, USA). Layers were separated by polyethylene frits (20 μm, Supelco, USA). A scheme of the assembled SPE cartridge is provided in ESM 1 (Fig. S2); the SPE steps are described in detail elsewhere [12]. Briefly, after conditioning (5 mL MeOH, 10 mL NPW) and sample loading, the cartridge was eluted upside-down with 6 mL alkaline (2% ammonia, *v*/*v*) and 3 mL acidic (1.7% formic acid, *v*/*v*) ethyl acetate/MeOH mixture (50:50, *v*/*v*) and finally with 2 mL MeOH. Eluates were combined, evaporated to 0.1 mL by nitrogen blow-down (40 °C), reconstituted in NPW to a final volume of 1 mL, and centrifuged at 3020*g* for 45 min at 20 °C (Megafuge 1.0R, Heraeus), before supernatants were transferred into amber glass LC vials. Resulting enrichment factors were 100 (IWW), 250 (EWW), and 1000 (NPW/SW).

### Instrumental analysis and data processing

SPE extract (15 μL) or VEC concentrate (100 μL), both corresponding to the same on-column sample volume (see ESM 1 Table S2), were injected into a polar RPLC C18 column (Atlantis T3, 3 × 150 mm, 3 μm; Waters, USA). NPW and MeOH, both acidified with 0.1% formic acid, were used as eluents for the chromatographic gradient from 5 to 95% MeOH in 17.5 min (see ESM 1 Table S3). Detection was achieved by HRMS/MS on a QExactive Plus mass spectrometer (Thermo Scientific, USA). Mass spectra were acquired in full-scan mode at a mass resolution of 140,000 (FWHM at *m*/*z* 200), with subsequent data-dependent MS2 (Top5, mass resolution 17,500). Separate runs were carried out for positive and negative ESI. TraceFinder (version 4.1 EFS, Thermo Scientific, USA) was used for automated targeted detection and integration of chromatographic analyte peaks by the ICIS algorithm at a mass tolerance of 5 ppm and with a minimum of three data points per peak. All integrations were reviewed manually. Inherent to the internal standard method, an IS was assigned to each analyte. Ideally, a matching IS was selected. If a matching IS was not available, an IS with a similar retention time (non-matching) was employed instead. Quantification was based on 1/*x*- or 1/*x*^2^-weighted linear or quadratic calibration curves generated by fitting analyte concentrations (*x*) against STD-to-IS peak area response ratios (RR, *y*), without forcing the fit through zero. To detect unknown compounds, raw data of interest was submitted to a Compound Discoverer (version 2.1, Thermo Scientific, USA) non-target workflow (see ESM 1 section S6 for details).

### Method comparison and validation

In the following sections, “workflow” refers to VEC or SPE with subsequent instrumental analysis. First, to enable comparison between VEC and SPE, absolute recoveries over the VEC/SPE step (AR-VEC/AR-SPE) and matrix effects during ESI of IS in VEC concentrates and SPE extracts (ME-ESI-VEC/SPE) were determined. Validation parameters determined for the (entire) VEC workflow were absolute recovery (AR-W), method limit of quantification (MLOQ), accuracy, and precision.

STD spike levels were adjusted to sample matrix (NPW/SW, 200 ng/L; IWW/EWW, 1000 ng/L), compound class (PFCs, × 0.1; x-rays, × 10), or were compound specific (metformin, 5-methylbenzotriazole, benzotriazole, caffeine, sweeteners: NPW, 200 ng/L; SW, 1000 ng/L; IWW/EWW, 5000 ng/L). 171 IS (80 ng of each, PFCs, × 0.1; x-rays, × 10) were added. If not stated otherwise, the determination of validation parameters was based on three (in certain cases two) replicates and the precision was estimated by error propagation.

#### Calibration, method quantification limits in NPW, accuracy, and precision

A 10-point STD calibration series was prepared over a mass concentration range from 0 (matrix blank) to 1000 ng/L (0, 0.1, 0.5, 1, 5, 10, 50, 100, 500, and 1000 ng/L; PFCs, ×0.1; x-rays, ×10) by the addition of STD and IS to 60 mL NPW in glass vials. This was followed by VEC and instrumental analysis. The MLOQ in NPW was determined as the lowest analyte concentration yielding a chromatographic peak of at least three data points in full-scan mode, with a signal-to-noise ratio greater or equal to 10, among at least two replicates, and a RR of at least twice the RR in the matrix blank. Analyte concentrations in SW (60 mL), EWW (15 mL), and IWW (6 mL) were quantified against the calibration series in NPW (60 mL), resulting in volume factors (VF) of 4 and 10 for EWW and IWW, respectively. To determine accuracy (spike recovery) and the associated precision (%RSD among ≥ 2 replicates), IWW, EWW, SW, and NPW were spiked with STD (spiked) or not (unspiked). STD and IS (all samples) were both added prior to VEC. Analyte concentrations in spiked and unspiked samples were then quantified. If the concentration in a spiked sample was at least twice the unspiked (background) concentration, and quantification was possible among at least two replicates, the accuracy was calculated according to Eq. (1) using average calculated analyte amounts and the respective VF.

1 $$ \%\mathrm{accuracy}\ \left(\mathrm{STD}\right)=\frac{\left(\mathrm{calc}.\mathrm{amount}\ {\left(\mathrm{STD}\right)}_{\mathrm{spiked}}-\mathrm{calc}.\mathrm{amount}\ {\left(\mathrm{STD}\right)}_{\mathrm{unspiked}}\right)\times \mathrm{VF}\times 100}{\mathrm{theoretical}\ \mathrm{spiked}\ \mathrm{amount}} $$

Analyte concentrations in unspiked environmental samples are reported in the “Application to environmental samples” section. Concentrations were only considered if MLOQs (“Absolute recovery of entire VEC workflow, method quantification limits in environmental matrices, and matrix effects during ESI” section) and accuracy were available, the latter to correct a concentration if a non-matching IS was assigned.

#### Absolute recovery of VEC and SPE step

To determine absolute analyte recoveries over VEC (AR-VEC) and SPE (AR-SPE), NPW and water samples (IWW, EWW, SW) were spiked with STD prior to VEC/SPE (pre), after VEC/SPE (post), or not at all (unspiked). In all cases, IS were added after VEC/SPE to fix RR and compensate for signal changes during subsequent instrumental analysis (primarily during ESI). Recoveries were calculated according to Eq. (2), inserting average RR among ≥ 2 replicates.

2 $$ \%\mathrm{AR}-\mathrm{VEC}/\mathrm{SPE}\left(\mathrm{STD}\right)=\left(\frac{\mathrm{RR}{\left(\mathrm{STD}\right)}_{\mathrm{NPW}\ \mathrm{or}\ \mathrm{matrix}}^{\mathrm{pre}}-\mathrm{RR}{\left(\mathrm{STD}\right)}_{\mathrm{NPW}\ \mathrm{or}\ \mathrm{matrix}}^{\mathrm{unspiked}}}{\mathrm{RR}{\left(\mathrm{STD}\right)}_{\mathrm{NPW}\ \mathrm{or}\ \mathrm{matrix}}^{\mathrm{post}}-\mathrm{RR}{\left(\mathrm{STD}\right)}_{\mathrm{NPW}\ \mathrm{or}\ \mathrm{matrix}}^{\mathrm{unspiked}}}\right)\times 100\% $$

#### Absolute recovery of entire VEC workflow, method quantification limits in environmental matrices, and matrix effects during ESI

Depending whether a matching or non-matching IS was assigned, absolute recoveries of analytes over the entire VEC workflow were calculated as analyte signal recovery (AR-W) according to Eq. (3) or (4) by comparison of either IS or STD peak areas in enriched sample matrices with respective peak areas in NPW. In either case, IS and STD were spiked prior to VEC. AR-W integrates effects of matrix constituents during sample manipulation, the concentration step (VEC), and instrumental analysis.

3 $$ \%\mathrm{AR}-\mathrm{W}{\left(\mathrm{STD}\right)}_{\mathrm{matching}\ \mathrm{IS}}=\left(\frac{\mathrm{average}\ \mathrm{peak}\ \mathrm{area}\ {\left(\mathrm{IS}\right)}_{\mathrm{matrix}}}{\mathrm{average}\ \mathrm{peak}\ \mathrm{area}\ {\left(\mathrm{IS}\right)}_{\mathrm{CAL}\ \mathrm{series}\ \mathrm{in}\ \mathrm{NPW}}}\right)\times 100\% $$

4 $$ \%\mathrm{AR}-\mathrm{W}{\left(\mathrm{STD}\right)}_{\mathrm{non}-\mathrm{matching}\ \mathrm{IS}}=\left(\frac{\left(\mathrm{peak}\ \mathrm{area}\ {\left(\mathrm{STD}\right)}_{\mathrm{matrix}}^{\mathrm{spiked}}-\mathrm{peak}\ \mathrm{area}{\left(\mathrm{STD}\right)}_{\mathrm{matrix}}^{\mathrm{unspiked}}\ \right)\times \mathrm{VF}}{\mathrm{peak}\ \mathrm{area}{\left(\mathrm{STD}\right)}_{\mathrm{NPW}}^{\mathrm{spiked}\ \mathrm{amount}}}\right)\times 100\% $$

MLOQs in environmental sample matrices were derived from MLOQs in NPW, AR-W and volume factors (“Calibration, method quantification limits in NPW, accuracy, and precision” section) according to Eq. (5).

5 $$ \mathrm{MLOQ}\left(\mathrm{matrix}\right)=\frac{{\mathrm{MLOQ}}_{\mathrm{NPW}}\times \mathrm{VF}}{\mathrm{AR}-\mathrm{W}\left(\mathrm{matrix}\right)} $$

Matrix effects (ME) during ESI of IS in VEC concentrates (ME-ESI-VEC) and SPE extracts (ME-ESI-SPE) were determined by Eq. (6) that compares average peak areas of IS post-spiked into environmental samples (“Absolute recovery of VEC and SPE step” section) with average peak areas of IS post-spiked into enriched NPW. A ME of 100% indicates no effect during ESI, a ME below 100% indicates ionization suppression, and a ME above 100% indicates ionization enhancement [25].

6 $$ \%\mathrm{ME}-\mathrm{ESI}-\mathrm{VEC}/\mathrm{SPE}\left(\mathrm{IS}\right)=\left(\frac{\mathrm{average}\ \mathrm{peak}\ \mathrm{area}\ {\left(\mathrm{IS}\right)}_{\mathrm{matrix}}^{\mathrm{post}\ \mathrm{VEC}/\mathrm{SPE}}}{\mathrm{average}\ \mathrm{peak}\ \mathrm{area}\ {\left(\mathrm{IS}\right)}_{\mathrm{NPW}}^{\mathrm{post}\ \mathrm{VEC}/\mathrm{SPE}}}\right)\times 100\% $$

## Results and discussion

### Substance selection

**Fig. 1:**
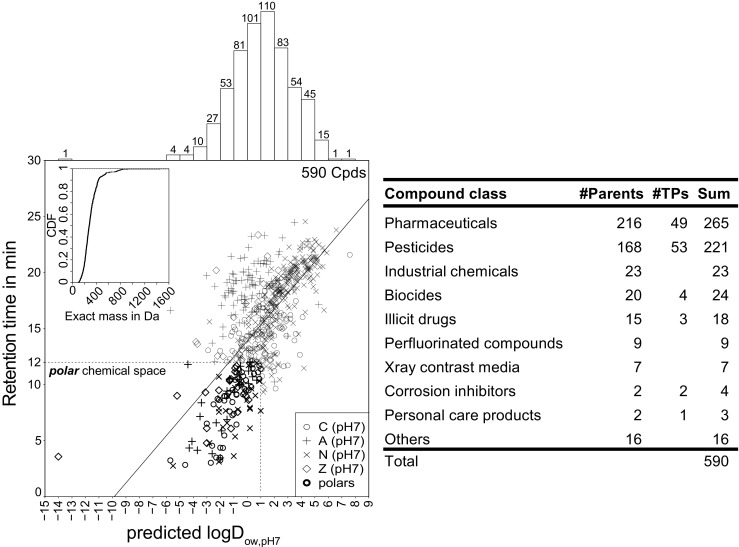
Substance selection. Left: chromatographic retention time (RT) versus predicted log*D* of the 590 organic contaminants selected for method validation. Symbols indicate the predicted major ion species at pH 7 either as cationic (C), anionic (A), neutral (N), or zwitterionic (Z). The *polar* chemical space includes 118 analytes with a log*D* ≤ 1 and a RT ≤ 12 min. Exact masses are displayed as cumulative distribution function (CDF) in the top left corner. The log*D* distribution is shown as histogram in the top left margin. Right: overview of number of parent compounds and transformation products (TPs) in different compound classes. See ESM 2 Table S6 for detailed substance properties

Five hundred ninety OCs (see ESM 2 Table S6) were selected to test and validate the suitability of VEC for the concentration of water samples prior to multiresidue trace organic analysis by LVI-polar RPLC-ESI-HRMS/MS. Substance selection criteria include environmental relevance, structural diversity, and physicochemical properties, i.e., to cover a wide range of analyte polarities (log*D*_ow,pH7_ − 14 to 8), different speciations (137 anionic, 130 cationic, 50 zwitterionic, 273 neutral), masses (102 to 916 Da, two analytes > 1000 Da), functional groups, and compound classes (Fig. 1, right). In the literature, polarity categories, such as *nonpolar*, *polar*, and *very* or *highly polar*, are often defined by different log*D*_ow_ ranges (e.g., [9, 11]). In this work, OCs with a predicted log*D*_ow,pH7_ ≤ 1 (JChem for Excel, version 18.8.0.253, ChemAxon) and a chromatographic retention time ≤ 12 min, i.e., approx. four times the column dead time, are considered *polar*. Hence, this classification forms a subset of 118 compounds (indicated in Fig. 1, left). In the following sections, “log*D*” always refers to log*D*_ow,pH7_.

### VEC workflow implications

VEC workflow fundamentals were adapted from an in-house mixed-bed multilayer SPE approach [12] such that enrichment factors and injection volume were adjusted to apply the same on-column sample volume (see ESM 1 Table S2). This approach facilitates a direct comparison of the two approaches. Prior to SPE, water samples are typically adjusted in pH and filtered. To minimize sample manipulation prior to VEC, reduce the risk of analyte loss, avoid contamination, and improve safe handling of sensitive analytes, samples were neither filtered nor adjusted in pH. Moreover, to ensure a soft evaporation process and to avoid thermal decomposition of analytes, the platform temperature during VEC was set to 55 °C. When a sample is evaporated to dryness, heat is no longer dissipated by evaporation, but directly transferred to the sample precipitate and ultimately to the analyte [26]. To avoid irreversible precipitation and heat stress on analytes, VEC was performed until roughly 0.3 mL (instead of evaporation to dryness) by means of a cooled (5 °C) glass vial appendix. Appendix cooling did not only ensure a residual volume but also potentially enhanced the stability of analytes in the VEC concentrate. By adjusting the concentrate to 0.4 mL, three injections (100 μL) were feasible and control over the final volume was established. As a last step before instrumental analysis, suspended particles were removed by centrifugation instead of filtration as there is only a small volume of purely aqueous VEC concentrate.

### Comparison of VEC and SPE

The subsequent sections discuss the extent to which analytes or analyte signals were affected. First, the differences between VEC and SPE as enrichment steps (“Absolute recoveries—VEC against SPE” section) are explained and then a detailed discussion on the presence of sample matrix within VEC concentrates and SPE extracts during ESI (“Matrix effects during ESI” section) is provided.

#### Absolute recoveries—VEC against SPE

Absolute recoveries derived for VEC and SPE, the associated precisions, and the number of compounds for which both could be calculated are summarized in Table 1 (see ESM 2 Table S6 for details). Reasons for non-computable recoveries include (i) a very high background concentration (RR in spiked sample were not at least twice the RR in the unspiked sample), (ii) complete analyte loss over SPE/VEC (peaks in post- but not in pre-spiked samples), (iii) no detectable peaks at all (neither in pre- nor post-spiked samples, hinting at a chromatographic issue), or (iv) irreproducible peaks (peaks were not detected among sufficient replicates). Taking the uncertainties of the analytical steps into account, an absolute recovery between 70 and 130% was considered acceptable. Most recoveries fall into this range (Table 1). In particular, median AR-SPE lies between 91 and 93% with a high precision between 3 and 5%. For AR-VEC, medians lie between 100 and 126% with the associated precision being slightly lower between 7 and 11%. High recoveries (167%) and the low precision in case of SW (32%) may be explained by the standard addition procedure, i.e., it was required for AR experiments to add IS (all samples) and STD (only post-spiked samples) after VEC in the lower part of the glass vials, followed by a manual vial wall rinse and evaporation until completion (0.3 mL). VEC resulted in the formation of precipitates (see ESM 1 Fig. S3), which was particularly extensive for SW samples. SW precipitates potentially promoted sorption, caused peak area variations among replicates and ultimately a lower precision and recoveries greater 130%. For example, IS peak areas in SW varied overall (median) by 25% when added after VEC compared to 10% when added before. In NPW, EWW, and IWW, this variation was observed infrequently (post vs. pre: NPW, 11% vs. 5%; EWW, 9% vs. 5%; IWW, 6% vs. 9%), as was the formation of precipitates. When the VEC workflow was applied for quantification of analytes in SW (“VEC validation parameters” section), precipitates did not interfere with the analysis, since IS were added prior to VEC. For recovery experiments with SW, a lower enrichment factor could be beneficial and reduce precipitate formation.

**Table 1:** Absolute analyte recoveries (median) and associated precisions (median) over the VEC and SPE step

Matrix	VEC			SPE		
	Number of compounds	Recovery in %	Precision as %RSD	Number of compounds	Recovery in %	Precision as %RSD
NPW	567	126	11	554	93	4
SW	539	167	32	533	93	4
EWW	551	102	7	542	91	3
IWW	525	100	7	520	91	5

Overall, SPE and VEC performed equally well (AR ≥ 70%) over all matrices for a large number of analytes (*n* = 327). Hence, the following sections will focus on OCs that were either especially or exclusively amenable to enrichment by either SPE or VEC.

**Fig. 2:**
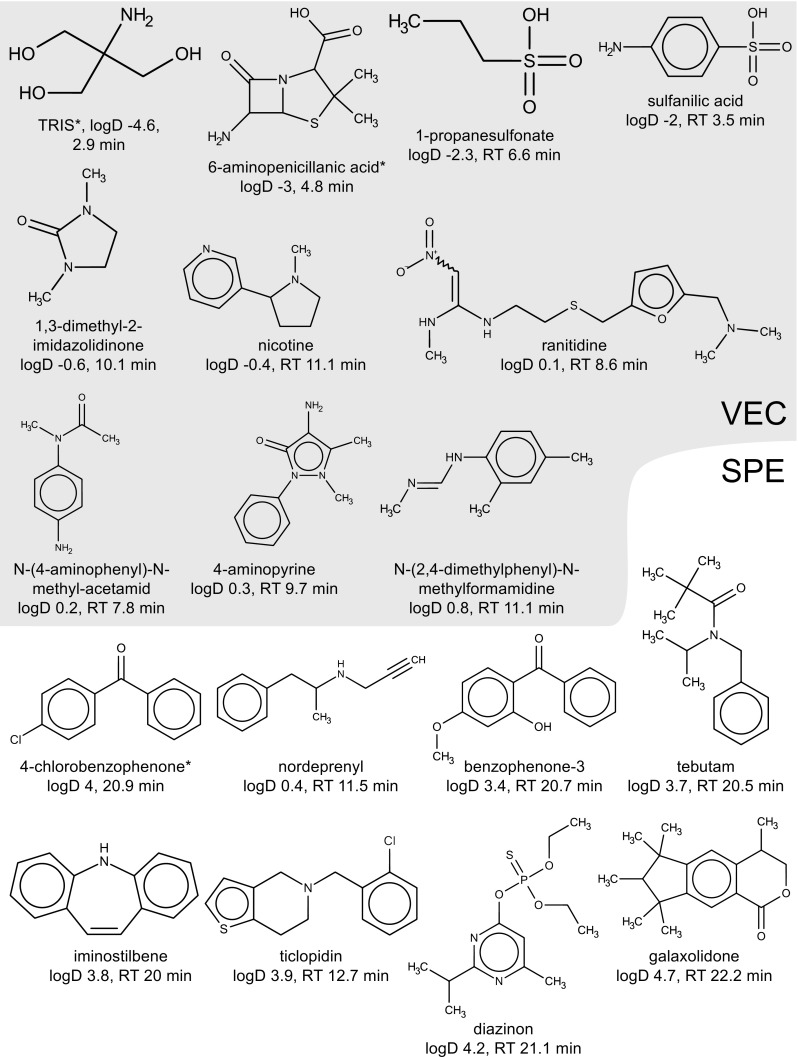
Analytes exclusively (indicated by asterisk) or especially amenable to enrichment by VEC (gray shade, only compounds of polar space shown) or SPE (polar space and nonpolar analytes) from all tested matrices. Molecular structures were created by MarvinSketch (version 18.8.0, ChemAxon) as part of the Jchem for Excel plugin (version 18.8.0.253, ChemAxon)

*Especially amenable* to VEC were ten analytes (eight shown in Fig. 2), most of them polar (median log*D* − 0.2, median RT 10 min): 1,3-dimethyl-2-imidazolidinone, 1-propanesulfonate, 4-aminopyrine, N-(2,4-dimethylphenyl)formamide, N-(4-aminophenyl)-N-methylacetamide, nicotine, ranitidine, sulfanilic acid, cilastatin, and cyazofamid. Their SPE recoveries were < 70% in all matrices, whereas their VEC recoveries were ≥ 70%. By contrast, seven analytes (Fig. 2), mostly nonpolar and either cationic or neutral (median log*D* 3.8, median RT 20.5 min) were more amenable to enrichment by SPE than VEC (VEC recoveries were < 70% in all matrices, while SPE recoveries were ≥ 70%): tebutam, diazinon, benzophenone-3, galaxolidone, iminostilbene, ticlopidine, and nordeprenyl.

Seven compounds were *exclusively amenable* to VEC and were mostly polar (median log*D* 0.2): TRIS, 6-aminopenicillanic acid, primaquine, perfluorohexanoic acid, lansoprazole, diazoxon, and pinoxaden. Their recoveries could only be determined for VEC (all matrices) but not SPE for several reasons (see above). An example is TRIS (Fig. 2), a polar OC with a log*D* of − 4.6 that was recovered by VEC from all matrices (median 115%) but lost during SPE. TRIS is an often used buffer substance that is expected to be removed during sample clean-up by SPE prior to LC-MS. By contrast, the only substance that was exclusively recovered by SPE (all matrices, median 85%) but lost during VEC (all matrices) was 4-chlorobenzophenone (Fig. 2), a nonpolar OC with a log*D* of 4. To investigate whether the loss of 4-chlorobenzophenone over VEC was related to its Henry’s law constant (HLC), HLC were estimated (25 °C, bond contribution methodology, see ESM 2) for 491 of the 590 organic substances using HenryWin (v3.20, embedded in EPI Suite v4.11, US EPA). No correlation between HLC and recovery over VEC or SPE became evident. However, the elevated HLC (17th highest of 491) of 4-chlorobenzophenone (1.4 × 10^−6^ atm m^3^/mol) could still be a possible explanation for its loss over VEC, but this remains speculative since very few even more volatile substances (according to estimated HLC) were recovered over VEC.

Considering the 118 analytes of the *polar* chemical space (log*D* ≤ 1, RT ≤ 12 min) separately, 110 were recovered by both workflows from at least one spiked matrix (including NPW), demonstrating the excellent performance of both workflows. For six polar analytes (see ESM 1 section 7; log*D* − 14 to − 3, RT < 5 min) recoveries could only be determined for VEC in NPW (lactitol, 2-amino-1,5-napthalenedisulfonic acid, acamprosat), few matrices (1,3-propylenediaminotetraacetic acid), or all matrices (Fig. 2; TRIS, 6-aminopenicillanic acid). 1,3-Propylenediaminotetraacetic acid was the most polar among the selected substances (log*D* − 14). It was recovered from VEC concentrates of all matrices except SW. However, it was neither detected in post- nor pre-spiked SPE extracts, suggesting a chromatographic issue related to SPE extracts (same for acamprosat and 2-amino-1,5-napthalenedisulfonic acid). By contrast, recoveries of two polar analytes could only be calculated for the SPE but not the VEC step (log*D* − 1.8 to − 1.9, RT < 6.2 min; NPW: allopurinol, SW: maleic hydrazide). The chromatography of selected polar OCs is shown in section S7 of ESM 1.

Of the 472 analytes *outside the polar chemical space* (log*D* > 1, RT ≤ 12 min or log*D* ≤ ≥ 1, RT > 12 min), 285 were equally amenable to both workflows (≥ 70% recovery) in all matrices, six especially to SPE, one exclusively to SPE (4-chlorobenzophenone) (all Fig. 2), two especially to VEC (cilastatin, cyazofamid; log*D* − 3.7 and 1.8, zwitterionic and neutral) and five exclusively to VEC (log*D* − 1.2 to 5.1; primaquine, perfluorohexanoic acid, lansoprazole, diazoxon, pionoxaden). VEC and SPE recoveries of the other 172 OCs were analyte-specific and matrix-dependent with values between 70 and 130% for all analytes (median) and matrices except SW (see above, precipitate interference).

*Overall*, the recovery data suggests the suitability of both workflows for a wide range of analytes. VEC appears especially suitable for the enrichment of polar compounds, with a few limitations regarding individual volatile or nonpolar ones for which SPE showed a better performance. The good SPE performance including polar analytes is attributed to the combination of diverse sorbent materials that were pre-selected and highly tuned for the simultaneous extraction of polar and nonpolar analytes. However, SPE based on a single sorbent material is by far the most widely used approach for the concentration of water samples prior to LC-MS [6]. As part of the in-house mixed-bed multilayer SPE method development [13], different sorbent materials, i.e., Oasis HLB, Strata X-AW/-CW, Isolute ENV+, and ENVI-carb™ were evaluated individually for analyte recoveries over SPE. In this context, 418 analytes were investigated, of which 380 overlap with the 590 substances selected for VEC validation. This allowed the comparison of analyte recoveries between Oasis HLB (a single sorbent), mixed-bed multilayer SPE based on multiple sorbents and VEC in NPW. The overall number of recovered analytes (VEC = multiple sorbents > HLB, 380 = 380 > 356) and the number of analytes recovered ≥ 70% (VEC > multiple sorbents > HLB, 358 > 331 > 273) indicate a clear benefit of the two latter methods over SPE with a single sorbent (Oasis HLB). Moreover, median log*D* of analytes recovered ≤ 70% (SPE < multiple sorbents < VEC, − 0.7 < − 0.1 < 3.3) and ≥ 70% (SPE > multiple sorbents > VEC, 1.6 > 1.2 > 1.0) emphasize and confirm the overall suitability of VEC towards polar analytes.

#### Matrix effects during ESI

**Fig. 3:**
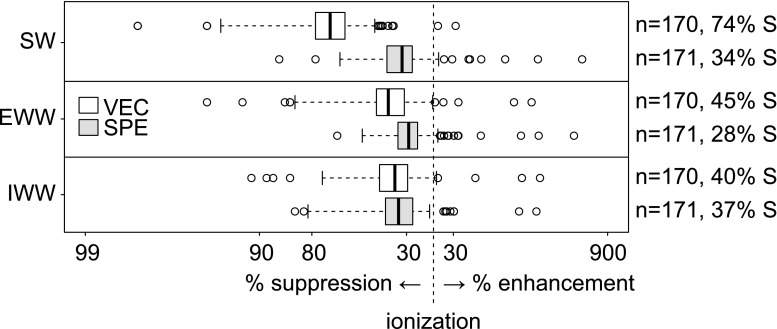
Matrix effects during ESI of IS in VEC concentrates and SPE extracts indicated as ionization suppression (S) and enhancement, i.e., | matrix effect − 100% |. Right margin: number of compounds, median over all compounds

Matrix effects (ME) during ESI were determined for 170 IS in VEC concentrates and 171 IS in SPE extracts for SW, EWW, and IWW (see ESM 2 Table S7). ME of IS in VEC concentrates strongly depended on matrix-specific enrichment factors. Specifically, SW had the highest enrichment factor (150×) and the median ME accounted for 26%, i.e., 74% ionization suppression (Fig. 3), followed by EWW (37.5×; ME, 55%) and IWW (15×; ME, 60%). In SPE extracts, the ME remained constant throughout the matrices with median values between 63 and 72%. For VEC concentrates of EWW and IWW, ME (EWW, 55%; IWW, 60%) and AR-W (“VEC validation parameters” section; EWW, 62%; IWW, 72%) were similar, suggesting that signal suppression during ESI is the likely cause of signal loss throughout the VEC workflow. Despite practical issues associated with SW (“Absolute recoveries—VEC against SPE” section), AR-W and ME were also similar (ME, 26%; AR-W, 28%), further emphasizing the overall role of signal suppression during ESI in the analysis of VEC concentrates.

### VEC validation parameters

**Fig. 4:**
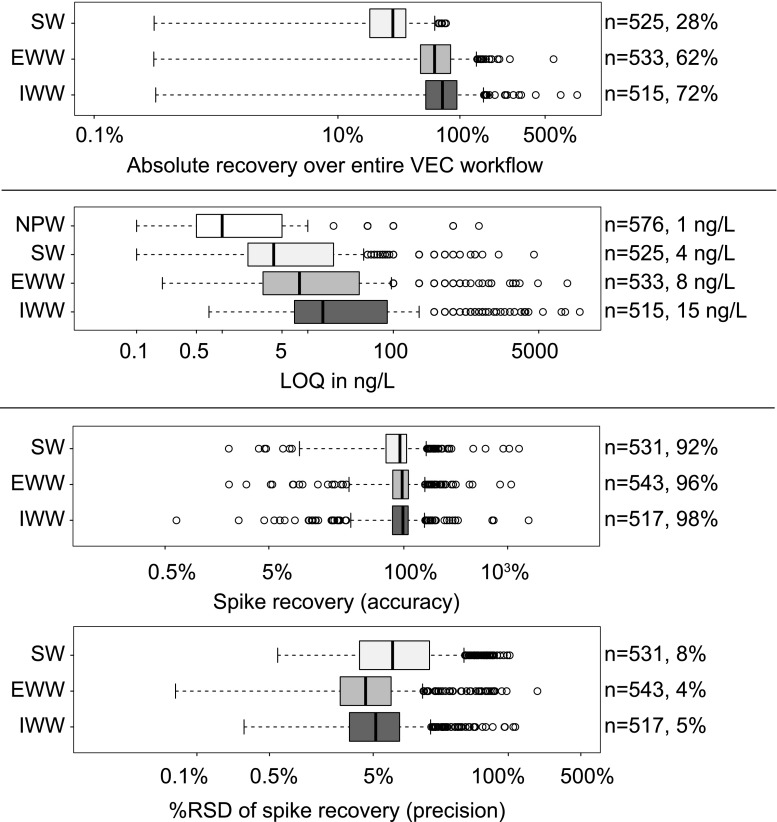
Validation parameters of the VEC workflow, i.e., absolute recoveries over the entire VEC workflow (AR-W) calculated as analyte signal recovery (top), method quantification limits (MLOQ, middle), accuracy, and precision (bottom). Right margin: number of compounds, median over all compounds

Absolute recoveries over the VEC *workflow* (AR-W, Fig. 4, top) could be calculated for 525 analytes in SW, 533 analytes in EWW, and 515 analytes in IWW. Interestingly, the assumingly simplest matrix (SW) caused the largest analyte signal loss over all workflow steps (smallest median AR-W of 28%), followed by EWW (62%) and IWW (72%). This can be explained by the increased enrichment of matrix with increasing enrichment factor (SW > EWW > IWW), accompanied by increasing analyte signal loss (decreasing AR-W). The increased enrichment of matrix was also reflected in the extent of precipitates formed in the bottom part of the glass vials as precipitates were more pronounced in SW than in IWW and EWW (see ESM 1 Fig. S3). For the analysis of SPE extracts by LC-ESI-MS/MS, AR-W is typically more similar for different matrices as compared to VEC. Obviously, during SPE (unlike VEC), analytes are not only enriched but are also extracted. Hence, the matrix interferences are removed (to a certain extent).

MLOQs in NPW, SW, EWW, and IWW show median values of 1, 4, 8, and 15 ng/L, respectively (Fig. 4, middle). Of the 576 MLOQs in NPW, 216 (38%) were at the sub-nanogram per liter level (˂ 1 ng/L), followed by SW (9%), EWW (8%), and IWW (1%). Furthermore, 360 of 576 OCs demonstrated a linear range in NPW from the respective MLOQ to the highest calibration level, i.e., a linear fit was applied. Linear ranges of 216 OCs ceased below the highest calibration level and a quadratic fit was more suitable. MLOQs were increasing (SW < EWW < IWW) with decreasing nominal enrichment factors (SW > EWW > IWW) and were higher than expected in SW since signal suppression was more pronounced than in the other matrices (EWW, IWW). In addition, the VEC workflow performed well in both aspects of accuracy (spike recovery in %) and precision (%RSD of spike recovery). Specifically, the median spike recoveries in SW, EWW, and IWW were close to 100% and precisions below 10% (Fig. 4, bottom).

### Application to environmental samples

**Fig. 5:**
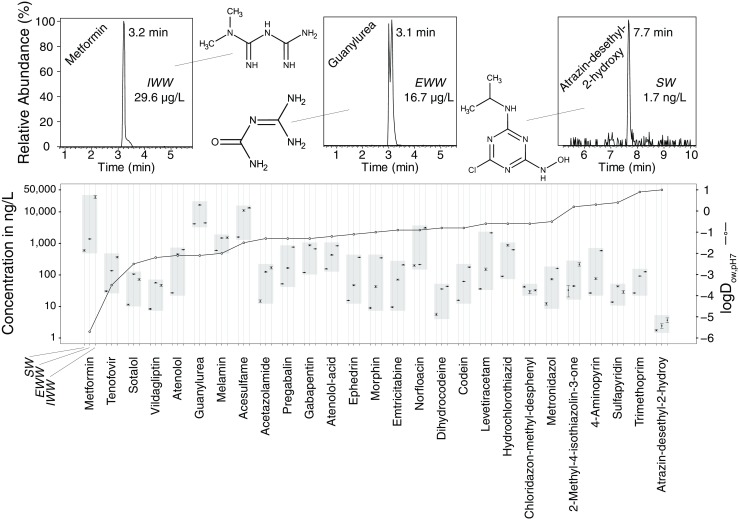
Concentrations (log scale) of polar analytes (log*D* ≤ 1, RT ≤ 12 min, sorted by log*D*) quantified in all environmental samples. Concentrations outside the calibration range: metformin (IWW), guanylurea (SW/EWW), acesulfame (SW/EWW/IWW). Guanylurea: peak splitting is a chromatographic artifact. Error bars indicate the standard deviation. Gray bars are a visual aid for grouping analyte concentrations

To further demonstrate the applicability to environmental samples, the 590 selected OCs (“Substance selection” section) were quantified in the unspiked SW, EWW, and IWW samples. One hundred twenty-one OCs were quantified in SW, 157 in EWW and 146 in IWW above the respective MLOQ. Of the quantified analytes, 51 belong to the *polar* chemical space (log*D* ≤ 1, RT ≤ 12 min) and 27 of these were detected in all matrices (Fig. 5). Of the analytes outside the polar chemical space, 73 (log*D* − 1.8 to 5.3, RT 10 to 22 min) were quantified in all matrices between 0.8 ng/L (N,N-didesmethylvenlafaxine) and 30 μg/L (caffeine, outside the calibration range). The least polar analytes were telmisartan, losartan, atazanavir, and propiconazole with a log*D* of 5.3, 5.1, 4.5, and 4.3, respectively.

### Applicability of VEC to non-target screenings

**Fig. 6:**
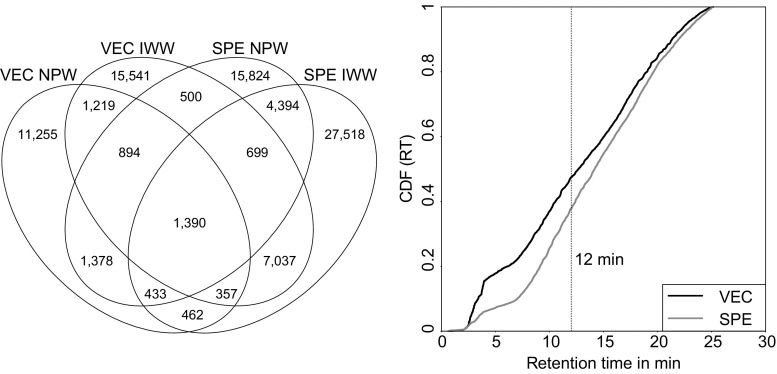
Comparison of compound numbers in NPW and IWW after enrichment via VEC and SPE (left). Right: cumulative distribution function (CDF) of retention times (RT) among compounds unique to VEC IWW concentrates (15,541) and SPE IWW extracts (27,518)

The use of HRMS does not only allow for the targeted analysis of known OCs but also enables the screening for suspected or unknown (polar) OCs, as well as (polar) TPs formed in the environment or lab-scale studies. The aim of a non-target screening is to detect unknown compounds that differ from background. Such screening was applied to VEC concentrates and SPE extracts of NPW, IWW, and EWW to identify the workflow that is more suitable for the concentration of water samples prior to a non-target screening. Unspiked NPW was analyzed to detect compounds originating from NPW itself and the analytical workflow (e.g., contamination from glassware, SPE materials) (Fig. 6, left). Less compounds were detected in VEC concentrates of unspiked NPW compared to the respective SPE extracts (VEC 17,388, SPE 25,512), hinting at the introduction of contamination throughout the SPE procedure. IWW samples were processed to identify the workflow that provided the larger number of compounds at a comparable overall ionization suppression (approx. 40% in SPE extracts and VEC concentrates; “Matrix effects during ESI” section). Similar to NPW, less compounds were observed in VEC concentrates of IWW samples (VEC 27,637, SPE 42,290). After blank subtraction (IWW minus NPW overlap), 23,777 compounds could be assigned to IWW VEC concentrates and 35,374 to IWW SPE extracts. Besides contamination, another potential explanation for this difference is that a considerable number of compounds in IWW SPE extracts were of nonpolar or volatile nature. Losses due to sorption to glass surfaces, precipitation, or volatilization during VEC are possible. To test this, compounds unique to VEC concentrates of IWW (15,541) and SPE extracts of IWW (27,518) were investigated for heteroatom content and retention time distribution. Heteroatom content among the suggested molecular formulae (VEC 76% by weight, SPE 71% by weight) and retention time distributions (47% of compounds in VEC IWW concentrates fall into the polar chemical space in terms of RT, i.e., ≤ 12 min compared to 38% of the compounds in SPE IWW extracts) (Fig. 6, right) both indicate that compounds unique to VEC IWW are more polar than compounds unique to SPE IWW. Thus, these results further suggest the potential and applicability of VEC for (non-target) screenings of unknown polar OCs or TPs.

## Conclusions and outlook

The developed VEC workflow is a valuable, environmentally friendly (minimal need for organic solvents) alternative to SPE that only requires minimal laboratory supervision at a lower cost. Its future application should be considered under the following conditions: (1) if analytes of interest are (very) polar while the LC in use is still performing well, (2) when the sample volume is limited, and/or (3) low LOQs are desired and not provided by direct sample injection, i.e., without enrichment. For the tested set of compounds, the VEC workflow performs exceptionally well despite using “only” a (polar) RPLC column. A mixed-mode LC column may improve the analyte retention further and expand the analytical space towards even more polar analytes. To exploit HILIC, VEC concentrates need to be made HILIC-compatible, requiring reconstitution in organic solvent (e.g., acetonitrile) at the expense of the enrichment factor. Alternatively, evaporation could be performed to dryness with subsequent reconstitution in organic solvent but at the expense of irreversible precipitation and loss of heat-sensitive (and volatile) analytes.

## Electronic supplementary material

ESM 1 (PDF 1.93 mb)

ESM 2 (XLSX 342 kb)
