# Supplementary material for: Vacuum-assisted evaporative concentration combined with LC-HRMS/MS for ultra-trace-level screening of organic micropollutants in environmental water samples
Source: Anal Bioanal Chem. 2019 Mar 11;411(12):2555–67. doi: 10.1007/s00216-019-01696-3 (PMC6470124; doi:10.1007/s00216-019-01696-3)
Supplement: Supplementary file 1 — (PDF 1.93 mb) [file 216_2019_1696_MOESM1_ESM.pdf]

Analytical and Bioanalytical Chemistry

Electronic Supplementary Material

Vacuum-assisted evaporative concentration combined with LC-HRMS/MS for ultra-trace-level screening of organic micropollutants in environmental water samples

Jonas Mechelke, Philipp Longrée, Heinz Singer, Juliane Hollender

Additional file available under https://doi.org/10.1007/s00216-019-01696-3.

Contents

S1 Scheme of the vacuum-assisted evaporation system and the mixed-bed multilayer SPE cartridge 3

S2 Pressure gradients applied during VEC 4

S3 On-column sample volume 4

S4 Chromatographic gradient 4

S5 Formation of precipitates 5

S6 Non-target screening 6

6.1 Compound Discoverer 2.1 – Workflow details 6

6.2 Applicability of VEC to non-target screenings 8

S7 Chromatography of selected polar analytes and their 8

7.1 Polar analytes exclusively amenable to enrichment by VEC from NPW 9

7.2 Polar analyte exclusively amenable to enrichment by VEC from NPW and few matrices ... 10

7.3 Polar analytes exclusively amenable to enrichment by VEC from all tested matrices 11

7.4 Polar analytes especially amenable to enrichment by VEC compared to SPE 12

References 16

S1 Scheme of the vacuum-assisted evaporation system and the mixed-bed multilayer SPE cartridge

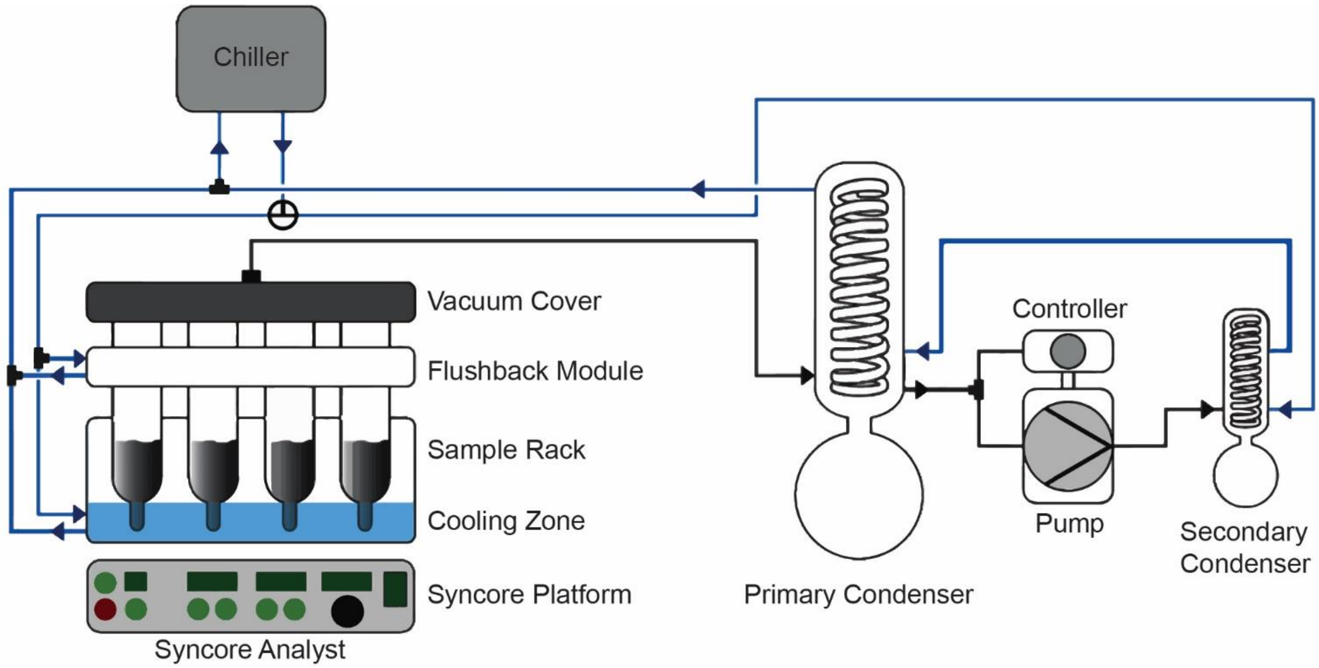

Fig. S1 Schematic setup of the vacuum-assisted evaporation system (Syncore Analyst, BÜCHI Labortechnik AG, Switzerland) including the vacuum pump and the chiller. *Blue lines*: cooling water loop [1]

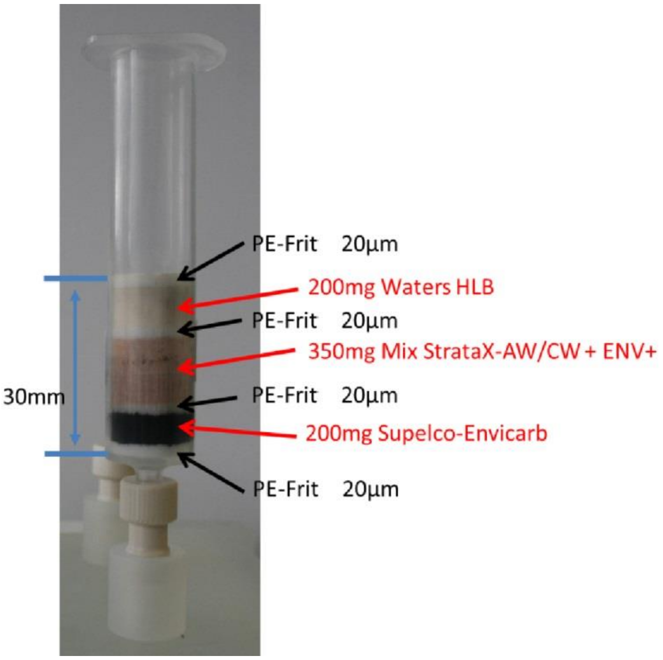

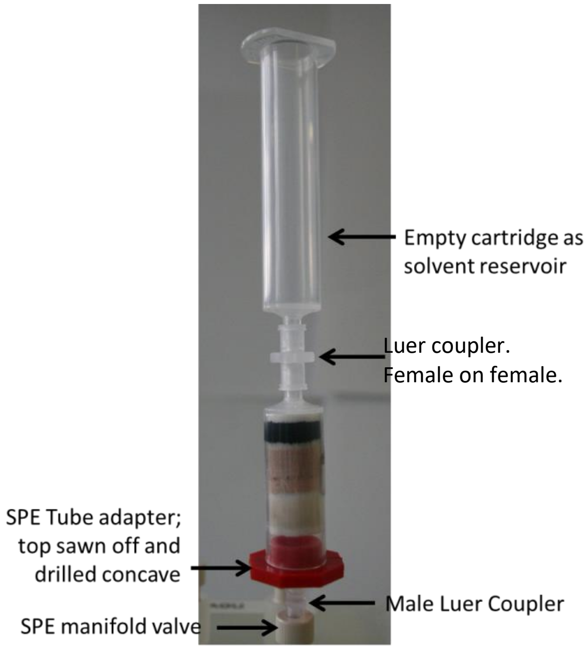

Fig. S2 Packed mixed-bed multilayer SPE cartridge. *Left*: loading position.
Right: cut-off cartridge for upside-down elution (back flush). *Photo credit*: Bernadette Vogler (Eawag)

S2 Pressure gradients applied during VEC

Table S1 Pressure (p) gradient for the automated (largely unattended) vacuum-assisted evaporative concentration of 60 mL (*left*) or 6 and 15 mL (*right*) aqueous sample

Step	p start [mbar]	p end [mbar]	duration [min]
1	1000	150	3
2	150	92	3
3	92	58	4
4	58	58	5
5	58	40	3
6	40	40	5
7	40	35	2
8	35	30	2
9	30	25	3
10	25	20	3
11	20	20	210

Step	p start [mbar]	p end [mbar]	duration [min]
1	1000	150	3
2	150	92	3
3	92	58	4
4	58	58	5
5	58	40	3
6	40	40	5
7	40	35	2
8	35	30	2
9	30	25	3
10	25	20	3
11	20	20	50

S3 On-column sample volume

Table S2 Overview on enrichment factors (EF), sample volume (V_s), final volume of concentrate (V_c), injection volume (IV), and the resulting on-column sample volume ($V_{on\text{-}column}$)

Matrix	**SPE**				**VEC**				$V_{on\text{-}column}$ [mL]
	V_s [mL]	V_c [mL]	EF	IV [mL]	V_s [mL]	V_c [mL]	EF	IV [mL]	
NPW	1000	1	1000	0.015	60	0.4	150	0.1	15
SW	1000		1000		60		150		15
EWW	250		250		15		37.5		3.75
IWW	100		100		6		15		1.5

S4 Chromatographic gradient

Table S3 Chromatographic gradient. *Eluent A:* NANOpure™ water acidified with 0.1% formic acid. *Eluent B:* methanol acidified with 0.1% formic acid

**Time**	**Eluent A%**	**Eluent B%**	**Flow rate [mL/min]**	**Detection**
0	95	5	0.3	yes
1.5	95	5		
17.5	5	95		
25	5	95		
25.5	95	5	0.3	no
29.5	95	5		

S5 Formation of precipitates

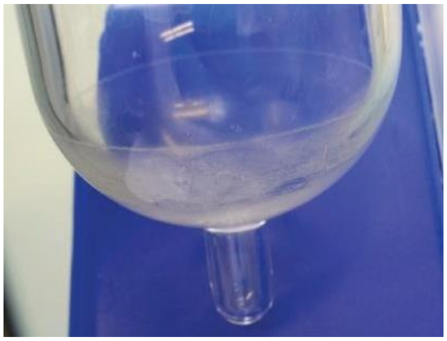

Fig. S3 Matrix precipitates after VEC exemplarily shown for effluent wastewater (enrichment factor: 20) during a preliminary experiment

S6 Non-target screening

6.1 Compound Discoverer 2.1 – Workflow details

The Compound Discoverer 2.1 (Thermo Scientific, USA) workflow is presented in Fig. S4. Detailed parameter settings are shown in Table S4.

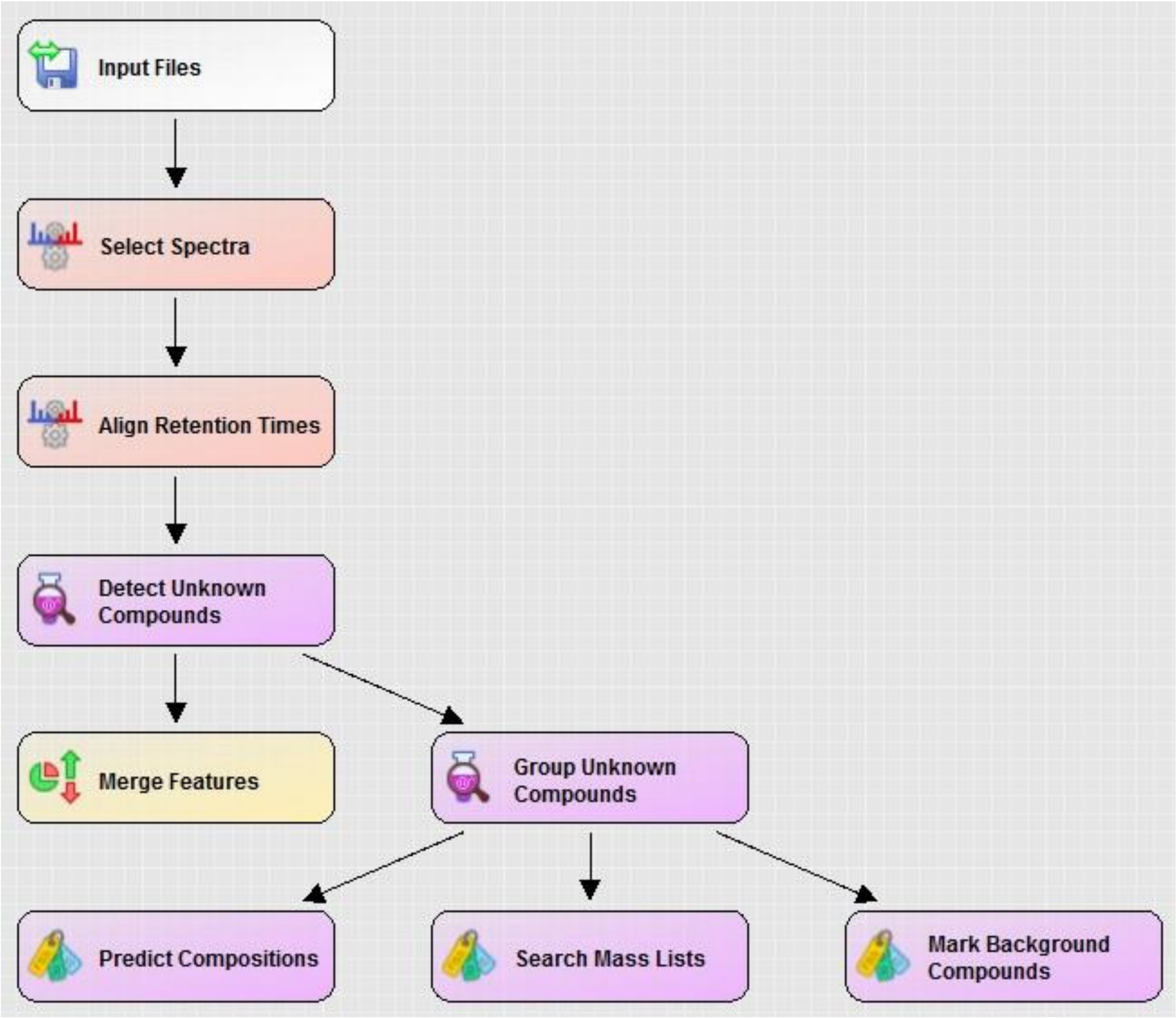

Fig. S4 Compound Discoverer 2.1 workflow diagram

Table S4 Compound Discoverer 2.1 workflow parameters

Processing Node	Applied Parameter Settings
Select Spectra	Presettings
	Polarity mode: + (pos batch), - (neg batch)
	Unrecognized Polarity Replacements: + (pos batch), - (neg batch)
Align Retention Times	Alignment Model: Adaptive curve
	Maximum Shift: 0.75 min
	Mass Tolerance: 5 ppm
Detect Unknown Compounds	Mass Tolerance: 5 ppm
	Intensity Tolerance: 30%
	S/N Threshold: 3
	Min Peak Intensity: 10000
	Preferred ions: pos ESI mode: [2M+H]+1; [M+2H]+2; [M+DMSO+H]+1; [M+H]+1; [M+K]+1; [M+Na]+1; [M+NH4]+1; neg ESI mode: [2M-H]-1; [M+Cl]-1; [M+FA-H]-1; [M-2H]-2; [M-H]-1
	Min Element Counts: C H
	Max Element Counts: C90 H190 Br3 Cl4 F6 K2 N10 Na2 O18 P3 S5
Group Unknown Compounds	Mass Tolerance: 5 ppm
	RT Toerance: 0.75 min
	Preferred ions: pos ESI mode: [2M+H]+1; [M+2H]+2; [M+DMSO+H]+1; [M+H]+1; [M+K]+1; [M+Na]+1; [M+NH4]+1; neg ESI mode: [2M-H]-1; [M+Cl]-1; [M+FA-H]-1; [M-2H]-2; [M-H]-1
Search mzCloud	Compound Classes: All
	Match Ion Activation Type: True
	Match Ion Activation Energy: Match with Tolerance
	Ion Activation Energy Tolerance: 30
	Apply Intensity Threshold: True
	Identity Search: HighChem HighRes
	Similarity Search: Similarity Forward
	Match Factor Threshold: 60
Mark Background Compounds	Max. Sample/Blanks: 3
	Max. Blank/Samples: 0
	Hide Background: FALSE
Search Mass Lists	Input files: \BU_VAL_ILISmasslist.csv
	Consider Retention Time: True
	RT tolerance: 2
	Mass Tolerance: 5 ppm
Predict Compositions	Mass Tolerance: 5 ppm
	Min. Element Counts: C H
	Max Element Counts: C90 H190 Br3 Cl4 F6 N10 O18 P3 S5
	Min. RDBE: 0
	Max. RDBE: 40
	Min. H/C: 0.1
	Max H/C: 3.5
	Max. # Candidates: 10
	Intensity Tolerance: 30 %
	Intensity Threshold: 0.1 %
	S/N Threshold: 3
	Use Dynamic Recalibration: True
	Use Fragments Matching: True
	Mass Tolerance: 10 ppm
	S/N Threshold: 3

6.2 Applicability of VEC to non-target screenings

Unknown compounds in effluent wastewater (EWW) samples were analyzed analogous to unknown compounds in IWW samples. Since the median ionization suppression was less pronounced in SPE extracts (28%) compared to VEC concentrates (55%), a fair comparison was not possible. Nevertheless, the respective compound numbers are presented in Table S5. The difference in ionization suppression was even greater for SW (VEC: 74%, SPE: 34%).

Table S5 Compound numbers in EWW and IWW after enrichment via VEC and SPE

**Total compounds**	**VEC EWW** 24‘909	**SPE EWW** 36‘334	**VEC IWW** 27,637	**SPE IWW** 42,290
Compounds after blank subtraction	20‘780	29‘337	23,777	35,374
Unique compounds	12‘742	21‘706	15,541	27,518
Heteroatom content (among total compounds)	79%	79%	73%	70%
Heteroatom content (among unique compounds)	82%	79%	76%	71%
Unique compounds in polar chemical space (RT ≤ 12 min)	49%	39%	47%	38%

S7 Chromatography of selected polar analytes and their

The following sections depict extracted-ion chromatograms (XICs, signal intensity versus retention time) of polar analytes ($logD_{OW,pH7}$ ≤ 1, RT ≤ 12 min) that were either exclusively recovered by VEC from NPW (section 7.1), by VEC from NPW and few but not all tested matrices (section 7.2), by VEC from all tested matrices (section 7.3) or polar analytes that were especially amenable to enrichment by VEC compared to SPE (all matrices, section 7.4). In the top margins, the values refer to analyte concentrations in ng/L, SWW, EWW and IWW refer to environmental samples to which the VEC workflow was applied for analyte quantification, i.e. surface water, wastewater effluent and wastewater influent, respectively, and R1 to R3 in the left margin indicate sample replicates. In each row, the first three XICs show the analyte signal in NPW (i) at the highest calibration level (1000 ng/L), (ii) in the matrix blank (‘0’ ng/L, only IS were added) and (iii) at the limit of quantification (LOQ). The LOQ in NPW was determined as the lowest analyte concentration yielding a chromatographic peak of at least three data points in full-scan mode, with a signal-to-noise ratio greater or equal to 10, among at least two of three replicates, and a STD-to-IS peak area response ratio (RR) of at least twice the RR in the matrix blank. Y-axes were scaled to the respective analyte signal intensity. In case of multiple chromatographic peaks, the correct peak is indicated by an arrow. Molecular structures in sections 7.1 and 7.2 were created using MarvinSketch (version 18.8.0, ChemAxon) as part of the Jchem for Excel plugin (version 18.8.0.253, ChemAxon).

7.1 Polar analytes <u>exclusively</u> amenable to enrichment by VEC <u>from NPW</u>

D-lactitol

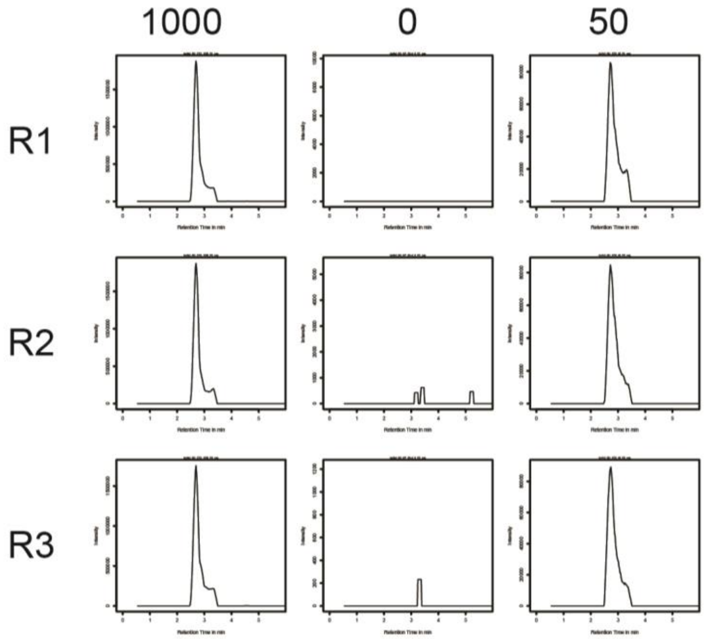

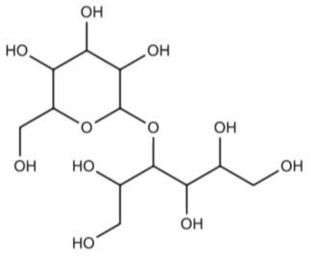

lactitol
logD -5.5, RT 2.8 min

2-amino-1,5-napthalenedisulfonic acid

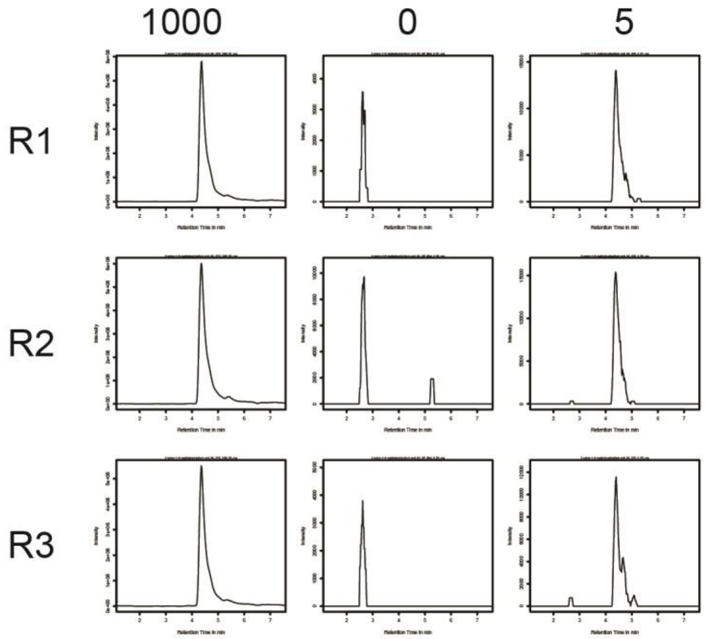

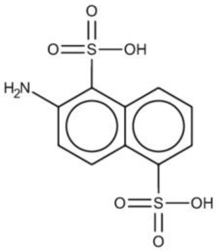

2-amino-1,5-naphthalenedisulfonic acid
logD -4.3, RT 4.4 min

acamprosat

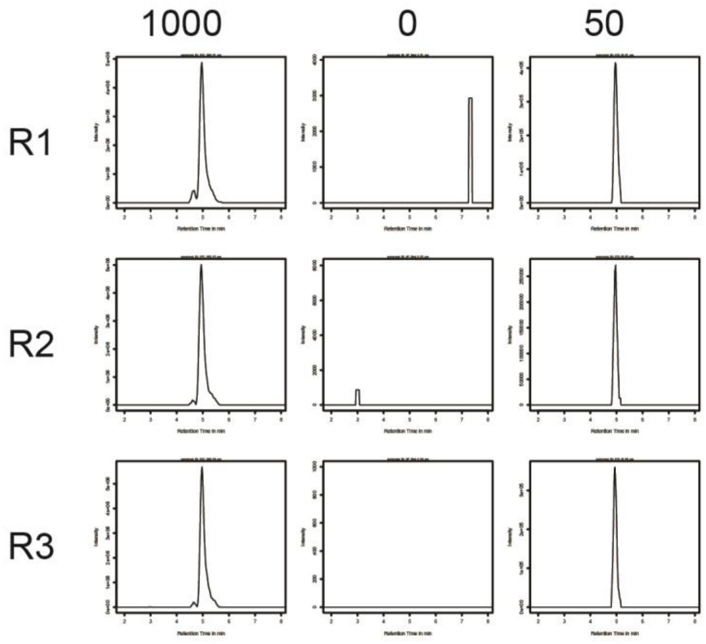

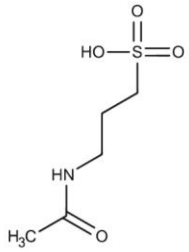

acamprosat
logD -4.1, RT 4.9 min

7.2 Polar analyte <u>exclusively</u> amenable to enrichment by VEC from <u>NPW and few matrices</u>

1,3-propylenediaminotetraacetic acid

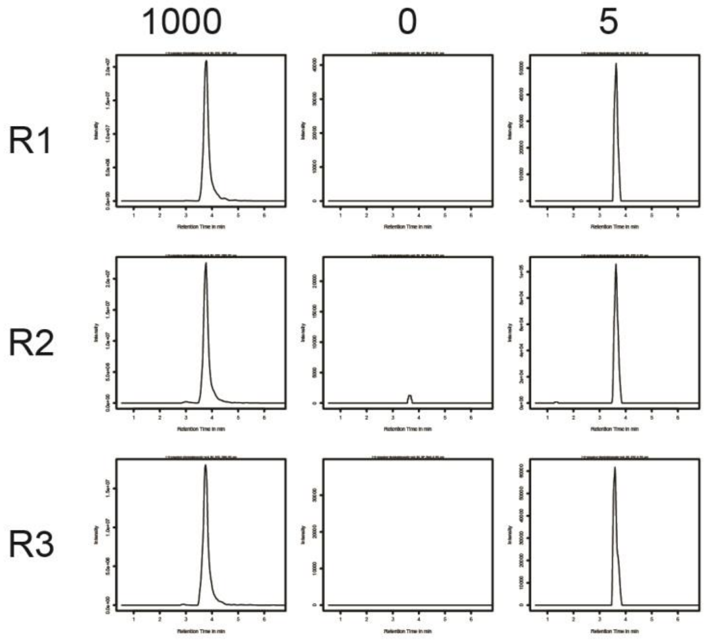

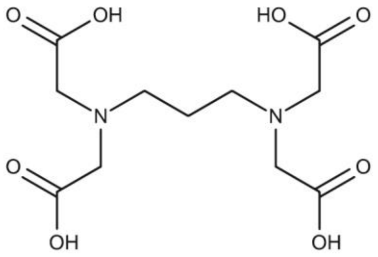

1,3-propylenediaminotetraacetic acid
logD -14, RT 3.6 min

7.3 Polar analytes exclusively amenable to enrichment by VEC from all tested matrices

6-Aminopenicillanic acid

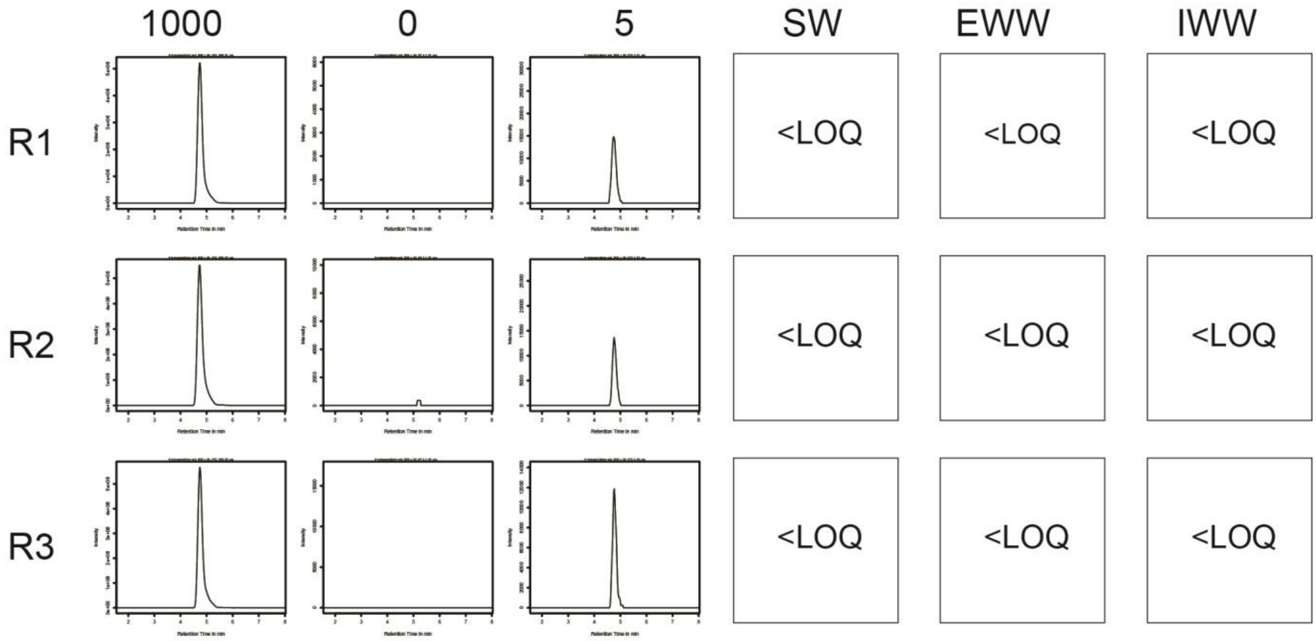

TRIS

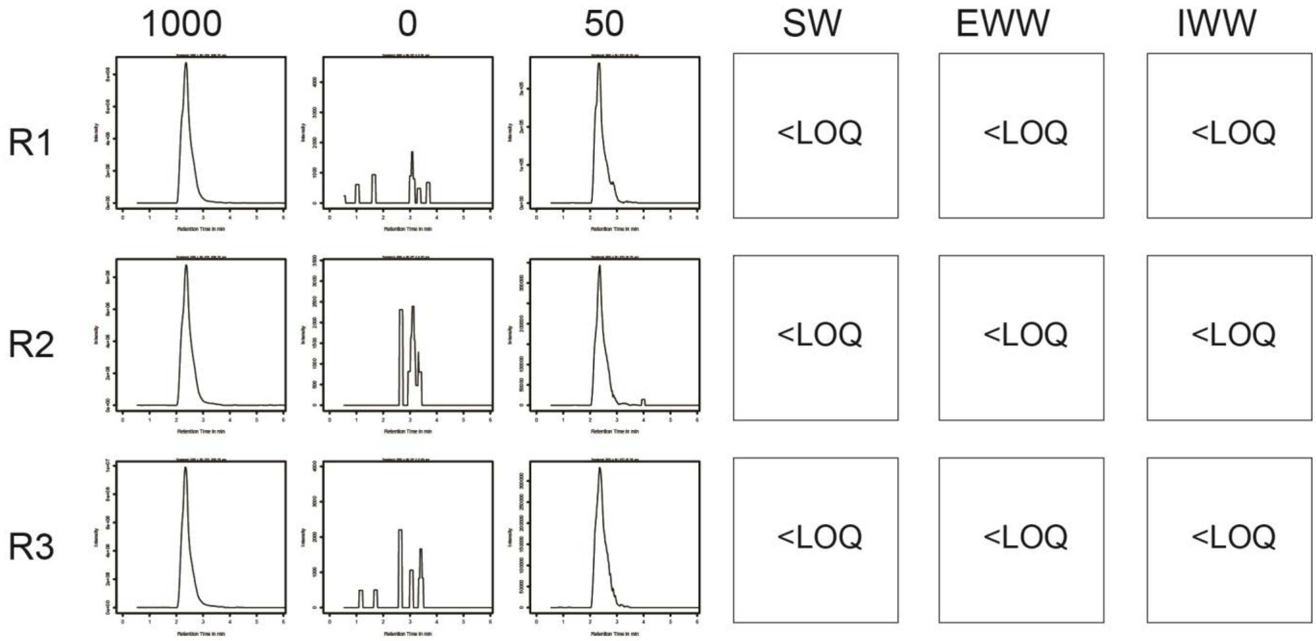

7.4 Polar analytes especially amenable to enrichment by VEC compared to SPE

1-Propanesulfonate

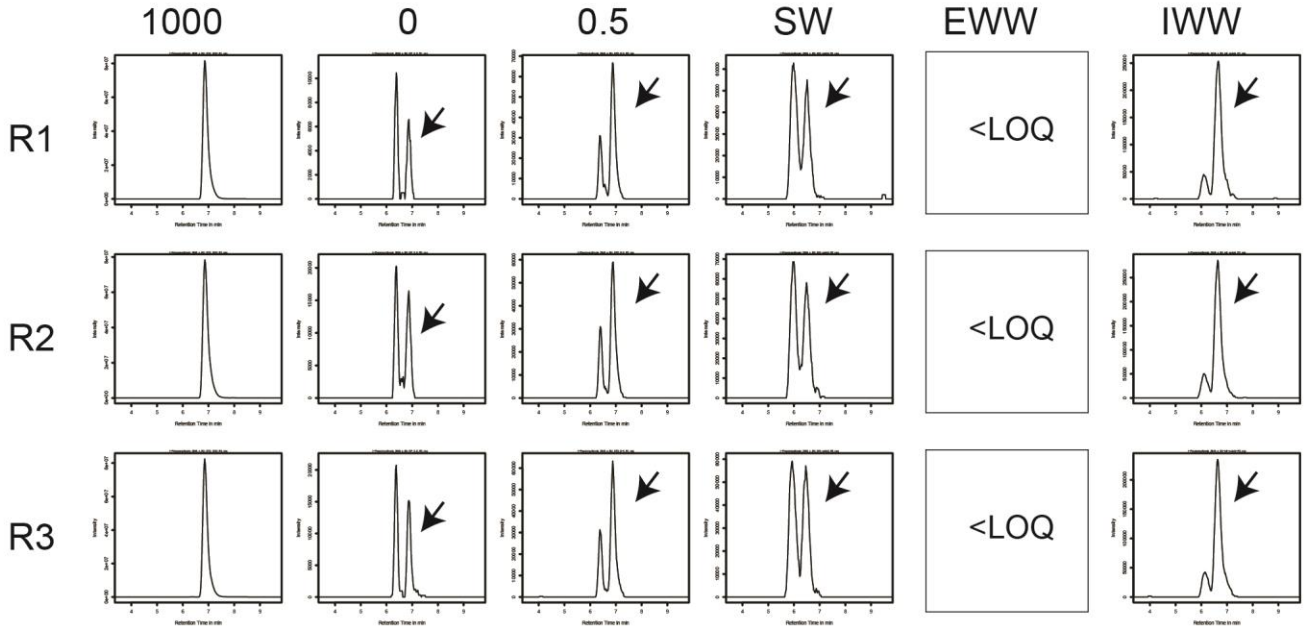

Sulfanilic acid

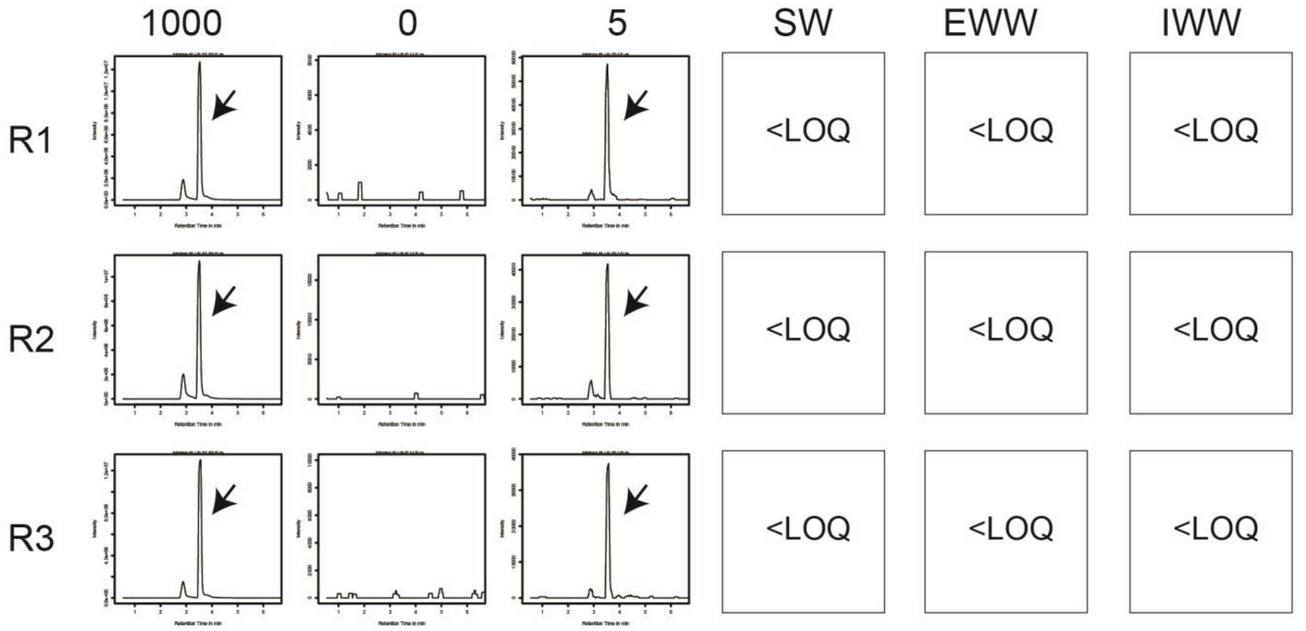

1-3-Dimethyl-2-imidazolidinone

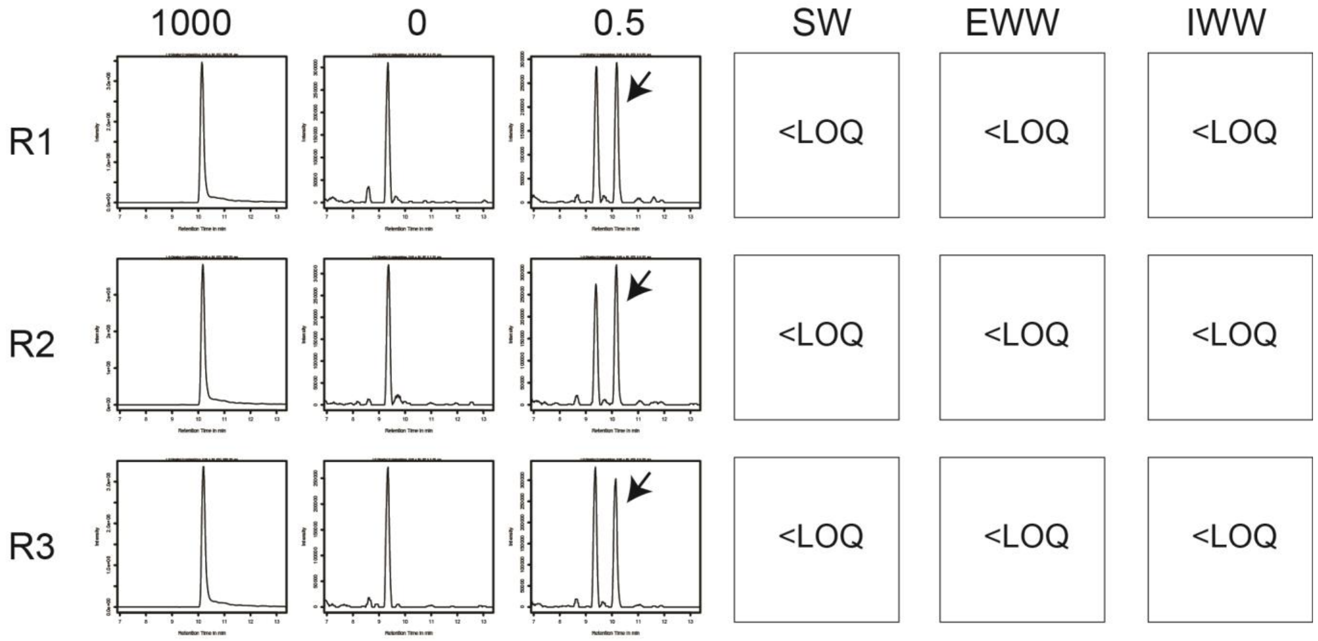

Nicotine

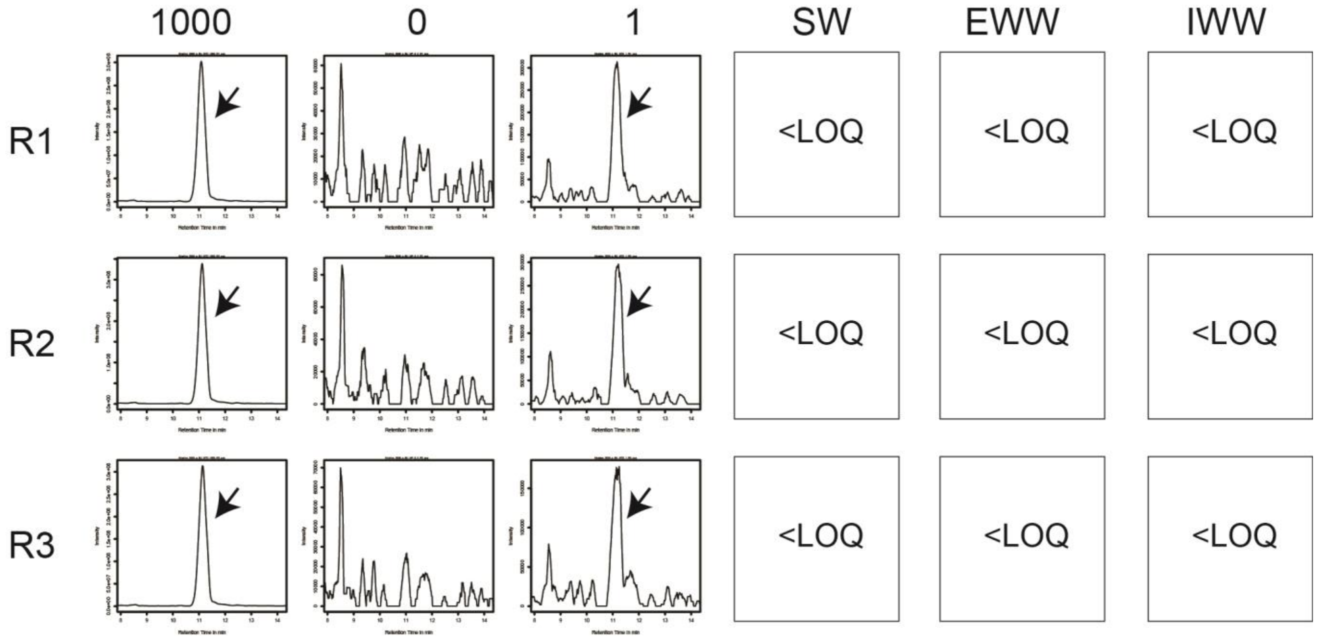

Ranitidine

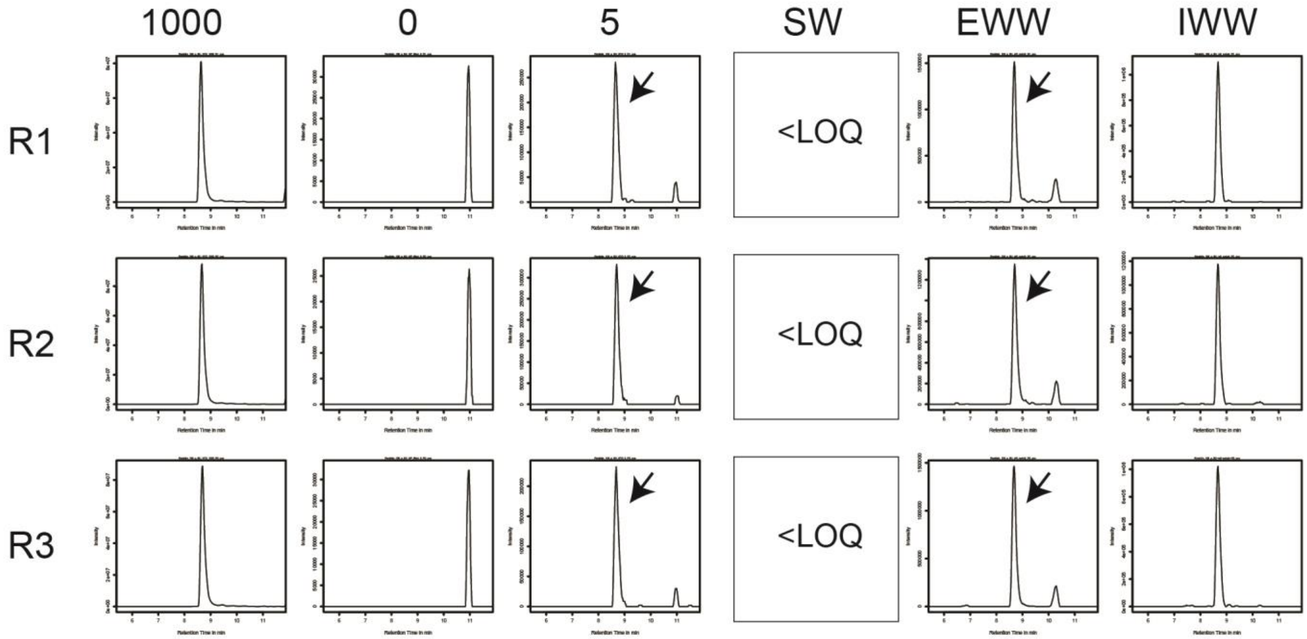

N-(4-Aminophenyl)-N-methyl-acetamide

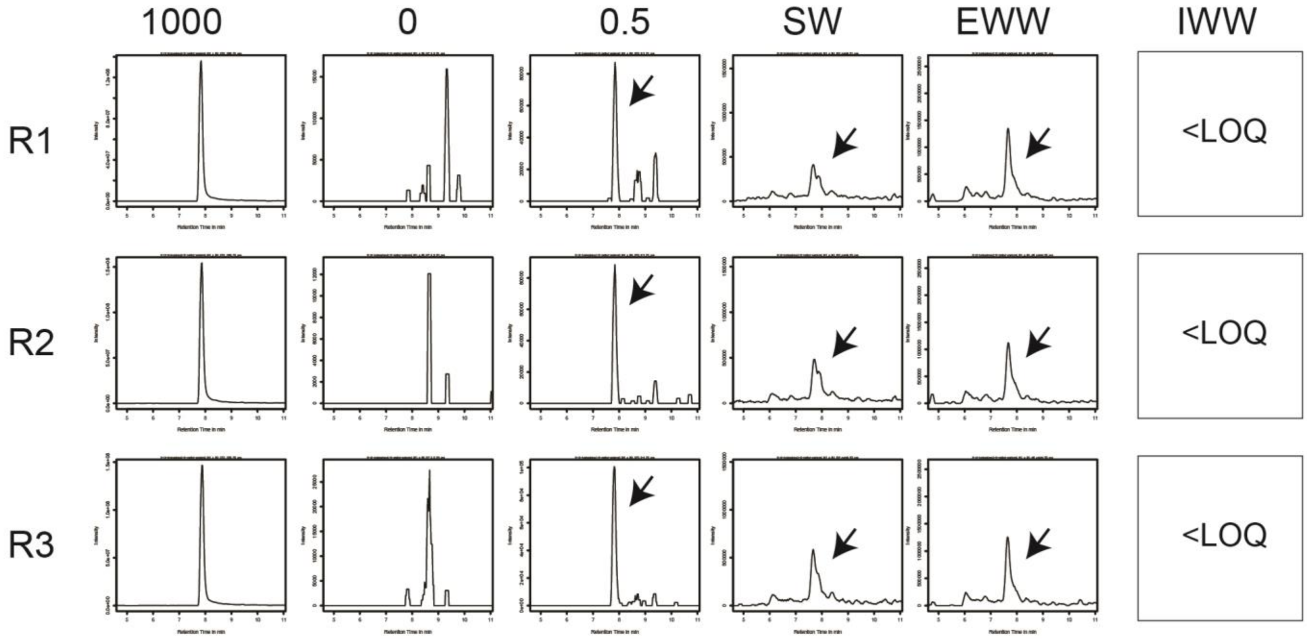

4-Aminopyrine (=4-Aminoantipyrine, 4-AA)

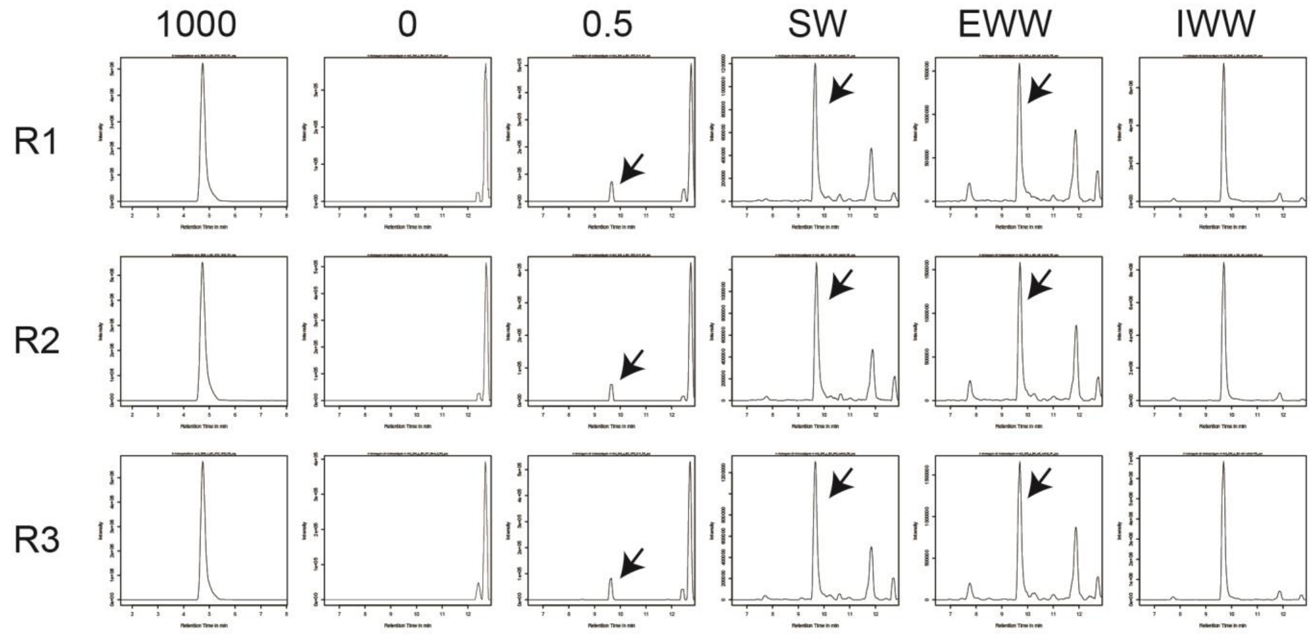

N-(2-4-dimethylphenyl)-N-methylformamidine

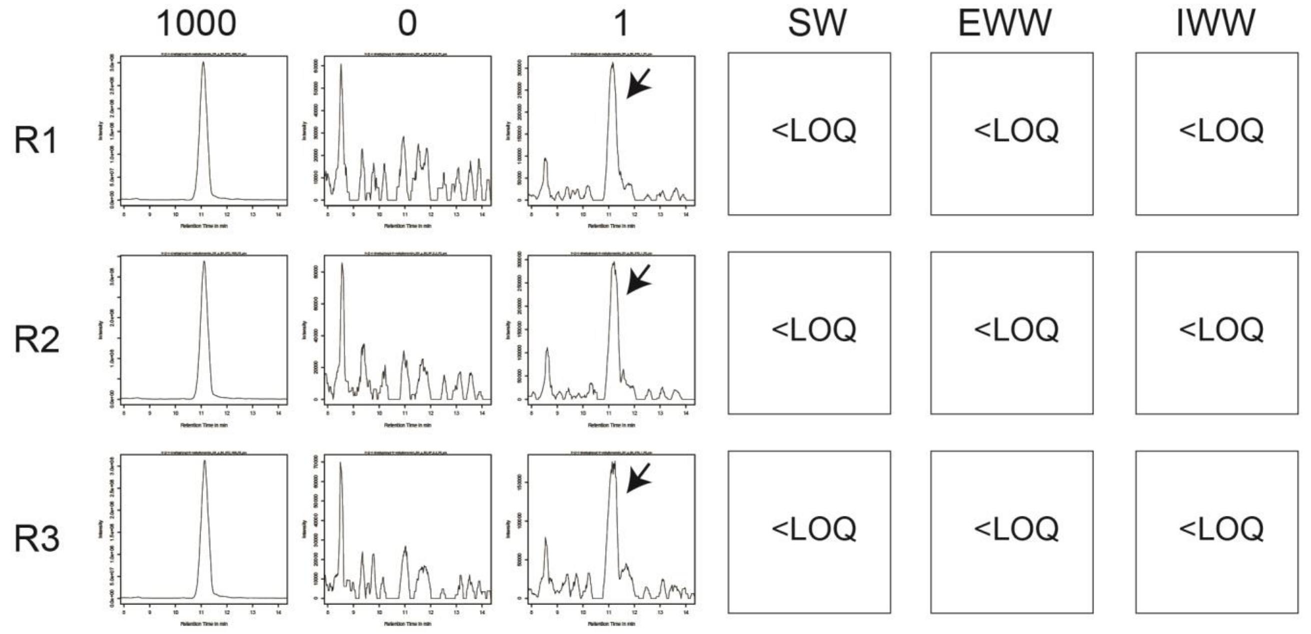

References

1. BUCHI Labortechnik AG (2012) Syncore Application Guide, Version A.
